# A transient disruption of fibroblastic transcriptional regulatory network facilitates *trans*-differentiation

**DOI:** 10.1093/nar/gku567

**Published:** 2014-07-10

**Authors:** Yasuhiro Tomaru, Ryota Hasegawa, Takahiro Suzuki, Taiji Sato, Atsutaka Kubosaki, Masanori Suzuki, Hideya Kawaji, Alistair R.R. Forrest, Yoshihide Hayashizaki, FANTOM Consortium, Jay W. Shin, Harukazu Suzuki

**Affiliations:** 1 RIKEN Omics Science Center, 1-7-22 Suehiro-cho, Tsurumi-Ku, Yokohama 230-0045, Japan; 2 RIKEN Center for Life Science Technologies, Division of Genomic Technologies, 1-7-22 Suehiro-cho, Tsurumi-Ku, Yokohama 230-0045, Japan; 3 Division of Genomic Information Resources, International Graduate School of Arts and Sciences, Yokohama City University, Yokohama 230-0045, Japan; 4 Discovery Pharmacology Department 1, Research Division, Chugai Pharmaceutical Co., Ltd, 1-135 Komakado, Gotemba, Shizuoka 412-8513, Japan; 5 RIKEN Preventive Medicine and Diagnosis Innovative Program, Wako, Saitama 351-0198, Japan

## Abstract

Transcriptional Regulatory Networks (TRNs) coordinate multiple transcription factors (TFs) in concert to maintain tissue homeostasis and cellular function. The re-establishment of target cell TRNs has been previously implicated in direct *trans*-differentiation studies where the newly introduced TFs switch on a set of key regulatory factors to induce *de novo* expression and function. However, the extent to which TRNs in starting cell types, such as dermal fibroblasts, protect cells from undergoing cellular reprogramming remains largely unexplored. In order to identify TFs specific to maintaining the fibroblast state, we performed systematic knockdown of 18 fibroblast-enriched TFs and analyzed differential mRNA expression against the same 18 genes, building a Matrix-RNAi. The resulting expression matrix revealed seven highly interconnected TFs. Interestingly, suppressing four out of seven TFs generated lipid droplets and induced PPARG and CEBPA expression in the presence of adipocyte-inducing medium only, while negative control knockdown cells maintained fibroblastic character in the same induction regime. Global gene expression analyses further revealed that the knockdown-induced adipocytes expressed genes associated with lipid metabolism and significantly suppressed fibroblast genes. Overall, this study reveals the critical role of the TRN in protecting cells against aberrant reprogramming, and demonstrates the vulnerability of donor cell's TRNs, offering a novel strategy to induce transgene-free *trans*-differentiations.

## INTRODUCTION

In any given cell, a set of transcription factors (TFs) is expressed and works in concert to govern cellular homeostasis and function (1). The Transcriptional Regulatory Networks (TRNs) in somatic cells are believed to be robust and act to prevent cells from aberrant plasticity (2). However, recent works have revealed that somatic cells in mammals are more plastic than previously thought (3). Particularly, Takahashi and Yamanaka demonstrated this notion by generating embryonic stem-like cells (or iPSC) from dermal fibroblasts after ectopic expression of Oct4, Sox2, Klf4 and cMyc (4,5). Additionally, increasing numbers of direct cell reprogramming, or *trans*-differentiations, toward neurons, hepatocytes and cardiomyocytes have also been reported (6–9).

Efficient *trans*-differentiations are believed to reset the TRN of the original cell type and re-establish the target TRN with a defined set of TFs (10–12). Although numerous examples have shown successful induction of target cell-specific functions and expression, most have largely ignored the involvement of the starting cell type, generally dermal fibroblasts. Several recent examples of (direct) cell reprogramming reported that a large number of fibroblast-specific genes and epigenetic marks remained intact even after inducing cell reprogramming (10–13). These reports suggest that the fibroblast TRN is highly robust and it may act to safeguard the cells from undergoing cell conversions. Therefore, how much the fibroblast-specific network plays a role in ‘inhibiting’ cell reprogramming remains to be elucidated.

To this aim, we first identified 18 fibroblast-enriched TFs based on the compendium of 988 human primary cells and tissues (FANTOM5) (14) and inferred a fibroblast TRN based on the Matrix-RNAi method (15–17). The analysis revealed inter-TF relationships including transcriptional activation/repression, directionality and combinatorial regulation of defined TFs and showed that 7 out of the 18 TFs were highly interconnected and influenced the majority of the fibroblast network. We further demonstrate that by targeting four out of seven major TFs, we could induce adipocyte-like cells in the presence of adipocyte medium without any ectopic expression of TFs. The global expression analysis revealed significant upregulation of adipocyte-specific marker genes and suppression of fibroblast-enriched genes. This study demonstrates the important inhibitory role of fibroblast-specific TFs in maintaining the fibroblast state and that the disruption of the underlying TRN can greatly enhance direct *trans*-differentiations. This work is part of the FANTOM5 project. Data downloads, genomic tools and co-published manuscripts are summarized at http://fantom.gsc.riken.jp/5/.

## MATERIALS AND METHODS

### Fibroblast-enriched TFs selection

Out of 988 CAGE human sample dataset (FANTOM5), 41 libraries are classified as fibroblasts from various tissues. To select fibroblast-enriched TFs from FANTOM5 dataset, the mean expression ratio of fibroblast was compared to the mean expression value of all non-fibroblast TFs in the dataset. All promoters with zero tag counts were set to 0.1. TFs for which expression ratio (fold-change) was above 1.5 were selected as candidates for fibroblast-enriched TFs. The human Illumina microarray dataset contained 14 human cell types: NB1RGB fibroblast, mammary epithelial cells (HMEC), keratinocytes (KER), renal epithelial cell (HRE), normal articular chondrocytes derived from the healthy knee joint (NHAC), prostate epithelial cells (PREC), osteocytes (OST), mesenchymal stem cell (MSC), skeletal muscle cells (SMC), MSC-ADP (MSC-derived adipocytes), CD14+ primary monocytes (MON), small airway epithelial cells (SAEC) and induced pluripotent stem (iPS) cells derived from NB1RGB. To select fibroblast-enriched TFs, NB1RGB fibroblast expression data were compared with the data of other 13 cell types one by one to calculate the expression ratio. TFs for which the average expression ratio in fibroblast was >1.5 were selected as candidates of fibroblast-enriched TFs. The intersect of TFs from the FANTOM5 data and Illumina microarray data was chosen as fibroblast-enriched TFs.

### Cell culture

NB1RGB, normal human neonatal skin fibroblast cells, and NHDF-Adult, normal human adult skin fibroblasts, were obtained from RIKEN Bioresource Center (Tsukuba, Japan) and Lonza (#CC-2511, Lonza, USA), respectively, and cultured in Minimum Essential Medium alpha (Wako, Japan) supplemented with 10% fetal bovine serum and penicillin/streptomycin (100 U/ml and 100 μg/ml; Invitrogen, USA) at 37°C in a 5% CO_2_. MSCs (#PT-2501, Lonza, USA) and pre-adipocytes (#PT-5020, Lonza, USA) were cultured and differentiated in accordance with manufacturer's instructions. See section ‘Adipogenic induction’ for details.

### siRNA transfection and RNA extraction

Fibroblast cells were seeded in 12-well cell culture plate (Nunc) at a density of 2 × 10^4^ cells/well one day before forward transfection was performed with 50 nM (final concentration) of each stealth small interfering RNA (siRNA), 2.5 μl of Lipofectamine RNAiMAX (Invitrogen, USA) and Opti-MEM (Invitrogen, USA) according to the manufacturer's instructions. Two or more stealth siRNAs (Invitrogen, Carlsbad, CA, USA) were tested for each target gene, and the one giving the highest level of knockdown was used for the Matrix-RNAi analysis. siRNA sequences are listed in Supplementary Table S4. Stealth RNAi negative universal control MED (Invitrogen) was used to calibrate siRNA transfection.

RNAs were extracted with RNeasy mini kit (QIAGEN, Germany) according to the manufacturer's instructions. RNA was quantified with NanoDrop (NanoDrop Technologies, USA). All siRNA transfection experiments were performed in biological quadruplicate.

### Quantitative real-time RT-PCR and Matrix-RNAi Analysis

Reverse transcription reaction was performed with PrimeScript RT-PCR Kit (Perfect Real Time, TAKARA BIO) and GeneAmp PCR System 9700 (Applied Biosystems, USA) according to the manufacturer's instructions. Quantitative real-time RT-PCR (qRT-PCR) was performed in 10 μl reaction mixture with SYBR Premix Ex Taq™ (Perfect Real Time, TAKARA BIO) on an ABI 7900 Fast Real-Time PCR System (Applied Biosystems, USA). PCR parameters consisted of heating at 95°C for 10 s, followed by 40 cycles at 95°C for 5 s and at 60°C for 30 s. The relative amount (expression ratio) of the target gene mRNA was normalized to the endogenous GAPDH mRNA using the 2^−ΔΔ^^CT^ methods. Standard deviation (SD) and *P*-value of ΔΔCT in a total of four biological replicates were calculated using a threshold to select significant transcriptional regulation edges. The cutoff threshold value was arbitrarily defined as 3 SD above the mean and *P*-value < 0.05. Only significant perturbations that passed the threshold value were used as reliable transcriptional regulation edges for depicting the Matrix-RNAi network. The networks were drawn using Cytoscape. The primer information is summarized in Supplementary Table S4.

### Adipogenic induction

Two days post siRNA transfection, culture medium was replaced with supplemented MEM alpha. One day later, transfected cells and MSCs were treated with adipogenic induction medium for three days (IBMX, indomethacin, dexamethasone, insulin; Lonza, PT-3102B/PT-4135) followed by maintenance medium for 4 days (insulin; Lonza, PT-3102A). The adipocyte induction regime was repeated for one more cycle (total two cycles).

### Lipid staining and quantification

Knockdown-induced adipocytes (KD-iADP) were generated in 12-well or 24-well plates. Two weeks post adipogenic induction, cells were rinsed with phosphate buffered saline (PBS) and fixed with 4% paraformaldehyde (Merck) in PBS. Lipid droplets and nucleus were stained with LipidTox (Invitrogen) and Hoechst 33342 (Invitrogen), respectively. Stained lipid droplets and nucleus were quantified using the Cellomics ArrayScan Reader XTi (Thermo Scientific). The experiment was repeated three times to assess reproducibility.

### Microarray analysis

First- and second-strand cDNA and cRNAs from 500 ng of total RNA were prepared using the Illumina TotalPrep RNA Amplification Kit (Ambion, USA). The concentration of cRNA was measured with NanoDrop (NanoDrop Technologies, USA) and the size distribution of cRNA was evaluated using the Agilent 2100 Bioanalyzer (Agilent Technologies, USA). For gene expression profiling, 500 ng of cRNA was employed for labeling and hybridization using HumanWG-6 v3 Expression BeadChip kit or HumanHT-12 v4 Expression BeadChip Kit according to the manufacturer's instructions. All microarray analysis was performed in biological triplicate. The Illumina microarray dataset was registered at Gene Expression Omnibus (http://www.ncbi.nlm.nih.gov/geo/) at NCBI as accession number GSE44439.

## RESULTS

### Selection of fibroblast-enriched TFs

Transcription Regulatory Networks (TRNs) are composed of constitutive and cell-specific sub-networks to coordinate biological functions and maintain cellular phenotype (1). In order to infer gene regulatory interactions in fibroblast cells we first identified fibroblast-enriched TFs based on the gene expression data produced by the FANTOM5 consortium (Functional Annotation Of the Mammalian Genome 5), in which 41 out of 988 samples were classified as fibroblasts from various tissues (14,18). The enrichment score for each TF was calculated based on the mean expression of a TF across 41 types of fibroblasts divided by the mean expression across 947 non-fibroblast samples. Fifty TFs showing a fold-change >1.5 and a *P*-value less than 0.05 were identified as the first set of fibroblast-enriched TFs (Supplementary Table S1). Since NB1RGB, a primary fibroblast cell line previously used for cell reprogramming (10–12), was missing from the FANTOM5 data, we used a similar strategy to select 44 fibroblast-enriched TFs that exhibited an enrichment score >1.5 (*P*-value < 0.05) between NB1RGB and 13 non-fibroblastic primary cell types based on our microarray expression (Supplementary Table S2). Eighteen TFs, which overlapped between the FANTOM5 selection and the microarray selection, were chosen for further analyses (Table 1).

**Table 1.** List of 18 fibroblast-enriched transcription factors based on FANTOM5 and microarray gene expression profiles

No.	Gene ID	Gene name	Symbol	Enrichment score (FANTOM 5)	Enrichment score (Microarray)
1	13345	Twist basic helix-loop-helix transcription factor 2	TWIST2	3.60	3.78
2	56956	LIM homeobox 9	LHX9	3.57	1.87
3	283078	Mohawk homeobox	MKX	3.23	2.79
4	5396	Paired related homeobox 1	PRRX1	3.00	3.08
5	51450	Paired related homeobox 2	PRRX2	2.67	3.66
6	4487	Msh homeobox 1	MSX1	2.52	2.62
7	7291	Twist basic helix-loop-helix transcription factor 1	TWIST1	2.51	1.98
8	130497	Odd-skipped related 1 (*Drosophila*)	OSR1	2.34	1.56
9	10370	Cbp/p300-interacting transactivator, with Glu/Asp-rich carboxy-terminal domain, 2	CITED2	2.30	2.90
10	862	Runt-related transcription factor 1; translocated to 1 (cyclin D-related)	RUNX1T1	2.23	2.00
11	297	Forkhead box D1	FOXD1	1.95	3.62
12	6926	T-box 3	TBX3	1.93	1.71
13	4784	Nuclear factor I/X (CCAAT-binding transcription factor)	NFIX	1.87	1.89
14	3215	Homeobox B5	HOXB5	1.64	2.39
15	3224	Homeobox C8	HOXC8	1.64	4.21
16	4212	Meis homeobox 2	MEIS2	1.63	2.26
17	3223	Homeobox C6	HOXC6	1.51	3.49
18	3221	Homeobox C4	HOXC4	1.51	2.20

### Building a fibroblastic TRN model by Matrix-RNAi

**Figure 1.**
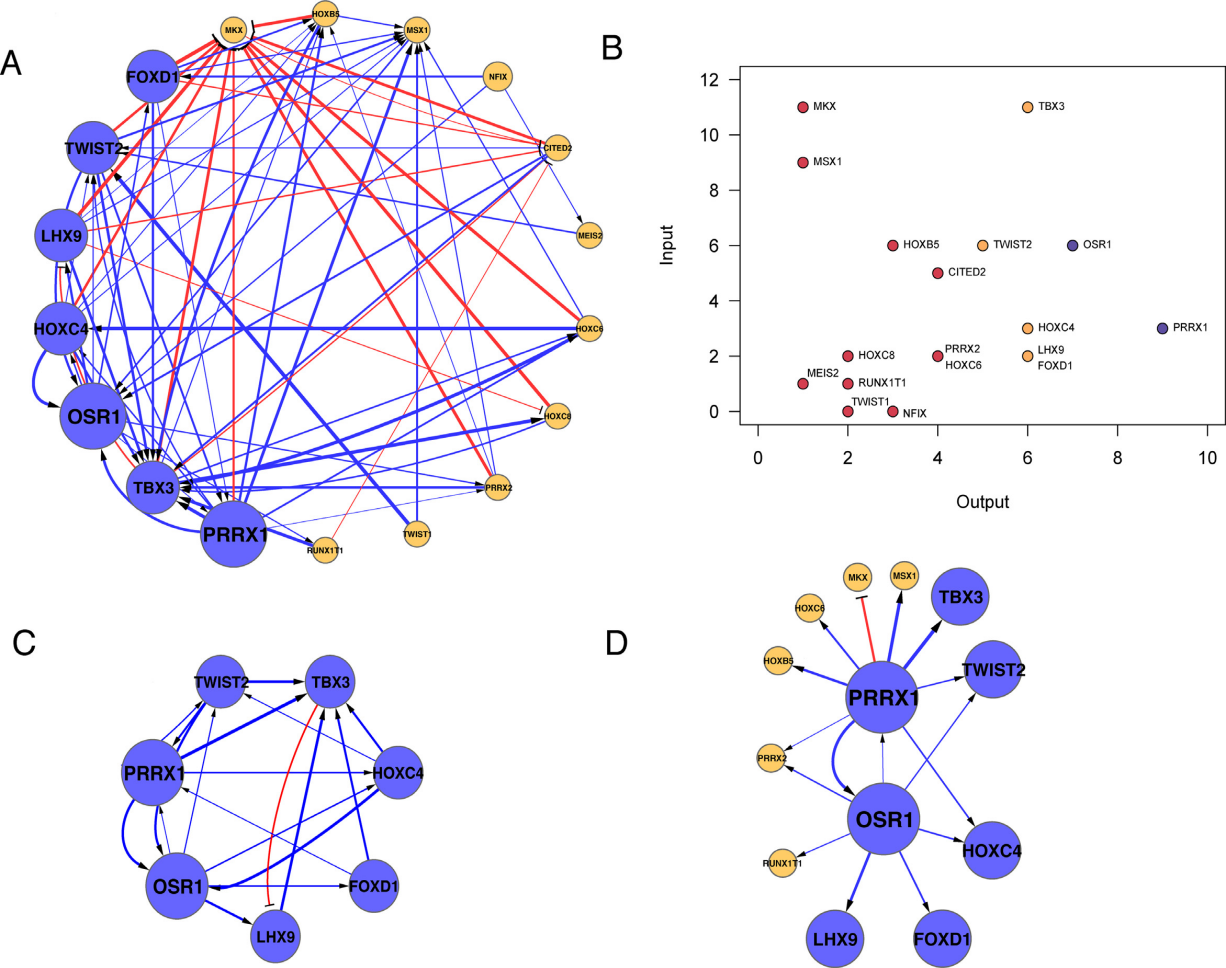
Establishment of fibroblastic transcriptional regulatory network (TRN) by means of Matrix-RNAi. (**A**) To reconstruct the fibroblastic TRN, edges with differential expression greater or less than 1.5 log-2 ratio (*P*-value < 0.05) were selected. Seven nodes with the highest regulatory edges were identified as influential TFs (blue nodes) and the remaining TFs were considered minor nodes (orange). Blue and red edges represent activating and inhibiting regulations, respectively. The thickness of the edges represents the strength of regulation based on the differential gene expression. (**B**) Scatter plot revealing the numbers of downstream (output) and upstream (input) target genes (four or less target genes in red; five or six target genes in orange; seven or more target genes in purple). (**C**) A selection of the seven major TFs demonstrating the dense interconnectivities. (**D**) The first neighboring nodes for the two TFs with the highest number of target genes, PRRX1 and OSR1, covering 11 out of 18 TFs. The sizes of the nodes indicate the number of edges per node.

In order to investigate the gene regulatory relationships among the 18 fibroblast-enriched TFs, we carried out the Matrix-RNAi perturbation analysis where each selected TF was systematically perturbed using siRNA. Subsequently, differential gene expressions of all 18 TFs were measured by means of quantitative RT-PCR (15,16), resulting in an 18-by-18 expression matrix (Supplementary Table S3). Based on the expression analysis, 72 regulatory edges were considered significant (*P*-value < 0.05 using Student's *t*-test). Fifty-three edges were activating and 19 were inhibiting (Figure 1A). The ratio of activating-to-inhibiting edges was similar to that of previously reported Matrix-RNAi in human hepatoma cell line, HepG2, and human monoblastic cell line, THP-1 (15,16).

TRNs are known to have hierarchical topology in discrete layers (19,20). In order to identify TFs with the most regulatory influence in the fibroblast network, the numbers of upstream and downstream target genes were quantified for each of the 18 TFs (Figure 1B). An influential-set, which consists of PRRX1, OSR1, FOXD1, HOXC4, LHX9, TBX3 and TWIST2, targeted five or more TFs in the fibroblast TRN. This influential-set explained 62.5% of the total number of regulatory edges and they collectively targeted 15 out of 18 TFs represented in the network. Upon a closer examination of the fibroblastic network, the interactions among OSR1–PRRX1–TWIST2 were notably coordinated, regulating one another in both directions, possibly working in concert to modulate downstream genes in fibroblasts (Figure 1C). Furthermore, the top two TFs with the highest number of target genes, PRRX1 and OSR1, collectively targeted all TFs in the influential-set, and covered 13 out of 18 TFs in the fibroblast network (Figure 1D). Retrospectively, six out of seven influential TFs were ranked among top 20 in the FANTOM5 list, while the seven TFs were evenly distributed in the microarray list (Supplementary Tables S1 and S2). This seems to suggest that the rich coverage of multiple cell types in the FANTOM5 dataset leads to a more informative set of TFs relevant to the maintenance of fibroblast network.

### Combinatorial TF-knockdown destabilizes fibroblast network and assists *trans*-differentiation toward adipocyte

**Figure 2.**
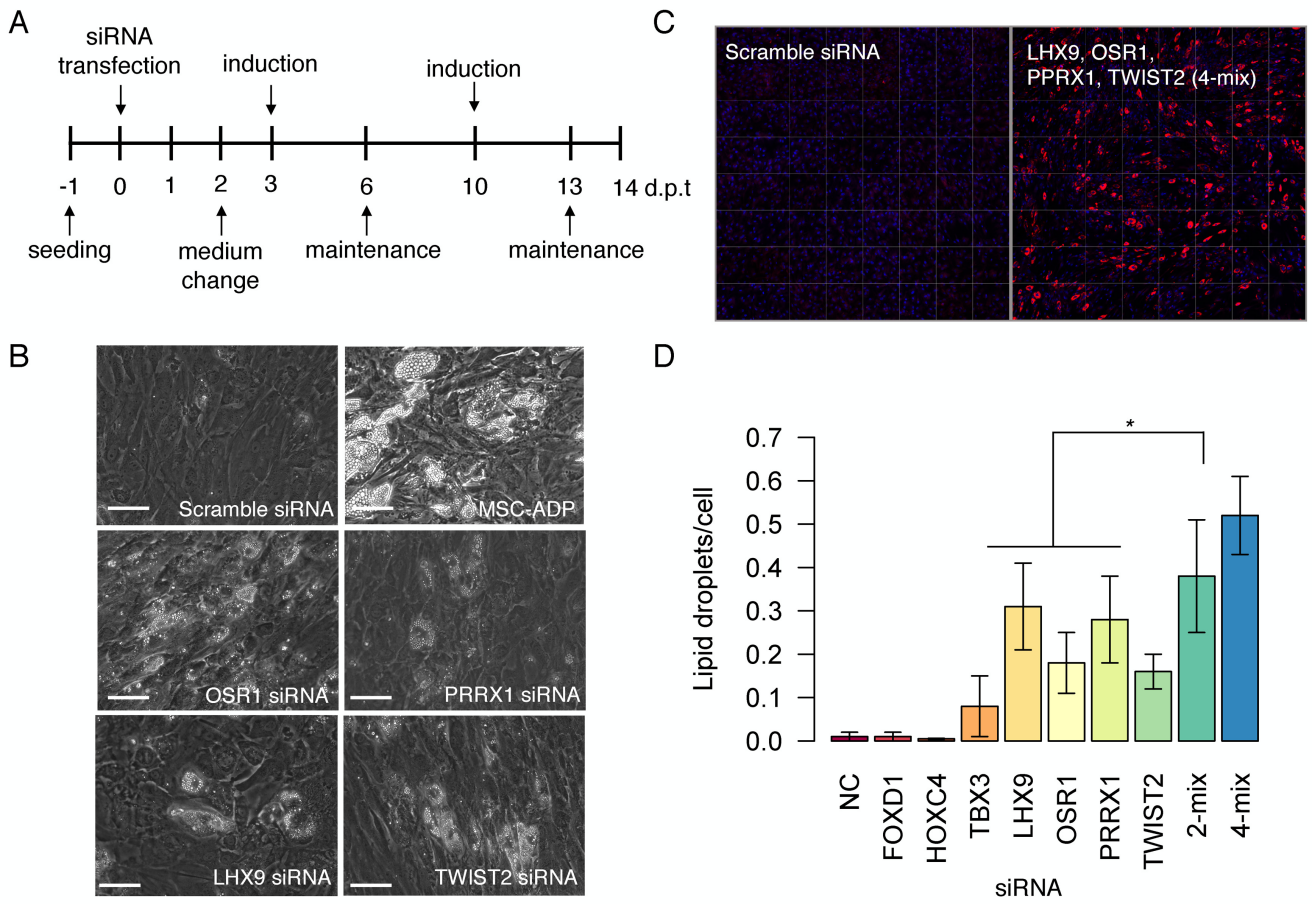
TF-knockdown promotes *trans*-differentiation from fibroblast to adipocyte. (**A**) Experimental workflow to generate adipocyte-like cells from fibroblasts by means of siRNA transfection followed by two cycles of adipogenic ‘induction’ medium and ‘maintenance’ medium. (**B**) Fibroblast cells transfected with scramble siRNA failed to generate lipid droplets while mesenchymal stem cells (MSCs) generated lipid droplets in the presence of adipogenic-induction medium. Knockdown of OSR1, PRRX1, LHX9 and TWIST2 induced adipocyte-like cells characterized by lipid accumulation. (**C**) For the quantification of lipids, cells were stained with LipidTox (red) and Hoescht (nucleus; blue). (**D**) The quantification of fluorescently labeled lipid droplets for single TF and combinatorial knockdown using the Cellomics ArrayScan image analysis software. 2-mix (OSR1 and PRRX1); 4-mix (LHX9, OSR1, PRRX1 and TWIST2). * denotes that 4-mix knockdown was statistically significant compared to individual TF transfections (Student's *t*-test *P*-value < 0.05). Scale bars = 50 μm.

Because multiple TFs mutually coordinate their activity to implement biological function and to maintain homeostasis (1), we reasoned that the combinatorial knockdown of a defined set of TFs might efficiently disrupt the fibroblastic TRN and broadly downregulate fibroblast genes. Such combinatorial knockdown would further decrease the stability of the fibroblast network where a chemical cocktail may provoke *trans*-differentiation into neighboring cell types. Since the protocols for adipogenic differentiation from MSCs and pre-adipocytes have been well established (21–23), we investigated the fibroblast-to-adipocyte transition after suppressing the fibroblastic network followed by repeated inductions of IBMX, dexamethasone, indomethacin and insulin, hereby referred to as adipogenic-induction medium (Figure 2A).

Adipogenesis is accompanied by a clear change in morphology along with the formation of lipid droplets, which can be observed under the standard phase-contrast microscope without additional preparation. In case of MSCs, a progenitor of adipocytes, the cells can efficiently differentiate into adipocytes possessing lipid droplets after 10 days of adipogenic induction medium (MSC-ADP; Figure 2B). In case of scramble siRNA transfected fibroblasts, no lipid formation was observed even when the cells were continuously stimulated with the same adipogenic medium (Figure 2B). Thereafter, we sought to test whether suppression of fibroblast network by means of siRNA knockdown can promote adipogenesis. All aforementioned 18 TFs were individually knocked down using siRNA followed by adipogenic-induction medium. After one week, we readily observed lipid droplet accumulations only when OSR1, PRRX1, LHX9 or TWIST2 was depleted (Figure 2B) but very few or no adipocytes were observed for the other 14 TFs (Supplementary Figure S1). Because TFs often work in concert to maintain TRN, we combined PRRX1 and OSR1 (2-mix) and PRRX1, OSR1, LHX9 and TWIST2 (4-mix) and transfected fibroblasts followed by adipogenic-induction medium. Remarkably, when we quantified the number of accumulated lipids based on fluorescent labeling (Figure 2C), we observed a significant increase in the number of lipid droplets in the 4-mix combination when compared to individual knockdowns (Figure 2D). Furthermore, when the 2-mix and 4-mix combinations were ectopically expressed in MSCs, the formation of adipocytes was significantly reduced when compared to the vector control (Supplementary Figure S2). These loss-of-function and gain-of-function analyses strongly suggest that the fibroblastic network plays an important role in ‘locking’ the cells in their native state, protecting the cells from aberrant transformations. To avoid any batch effects, we could also induce lipid droplet accumulation in dermal fibroblasts derived from a different individual (normal human dermal fibroblasts—adult; Supplementary Figure S3).

### Gene expression profile of adipocyte-like cells

**Figure 3.**
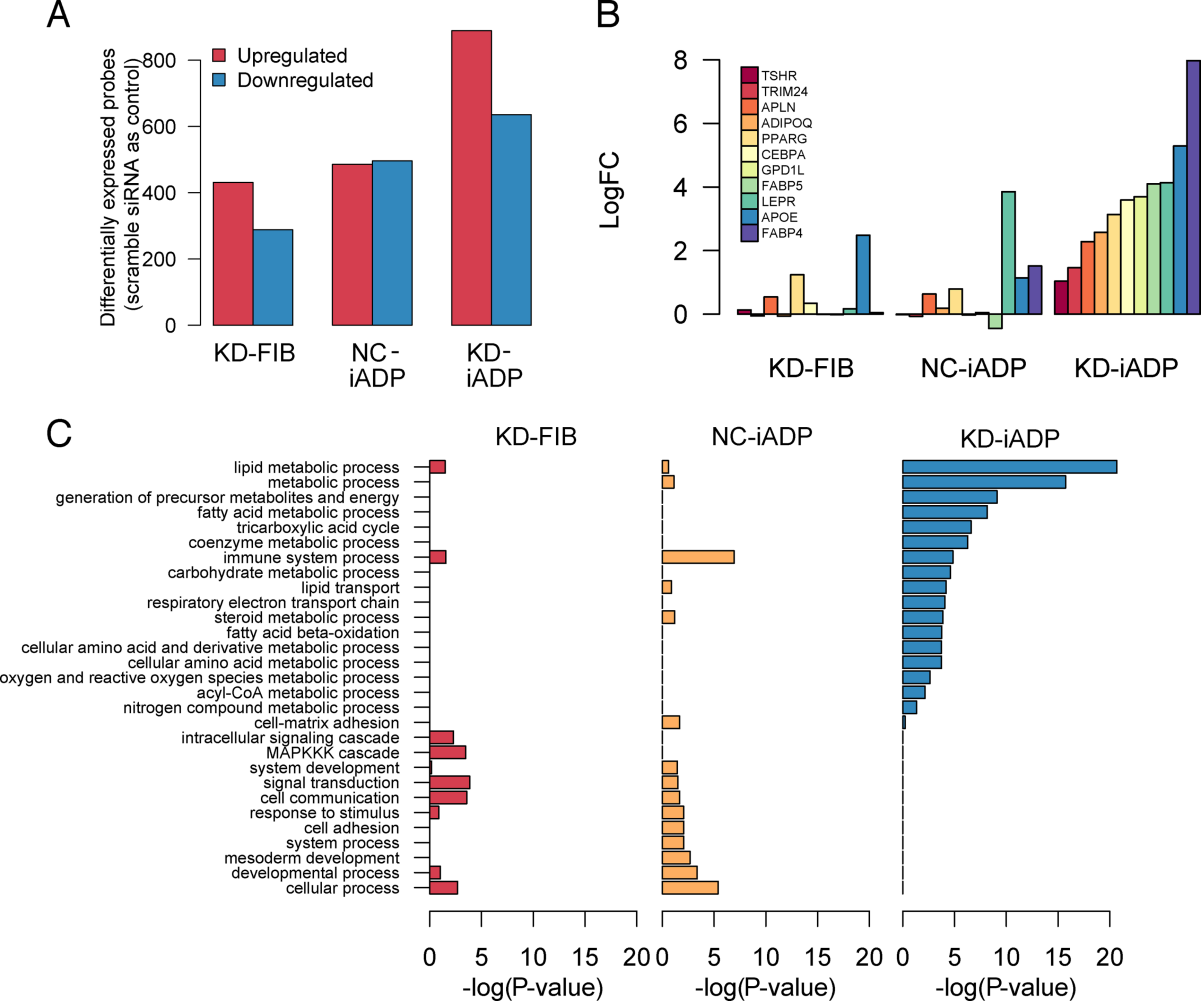
Genome-wide transcriptome expression analysis of knockdown-induced adipocytes. (**A**) Differentially expressed Illumina microarray probes after TRN knockdown (KD-FIB) and adipogenic induction in fibroblasts (NC-iADP) and both TRN knockdown and adipogenic induction (KD-iADP; Benjamin–Hochberg multiple testing *P*-value < 0.05). (**B**) Induction of adipocyte-specific marker genes in KD-FIB, NC-iADP and KD-iADP. Each gene was independently validated by qRT-PCR. (**C**) Gene ontology analysis reveals increase in gene expression involved in adipocyte functions such as lipid, fatty acid and steroid metabolic processes in KD-iADP 2 weeks post transfection.

To further characterize the 4-mix KD-iADP, we performed genome-wide transcription profiling to explore the global gene expression patterns of TF-suppressed fibroblasts with/without adipogenic-induced medium. The number of differentially regulated genes was significantly larger in KD-iADP cells when compared to cells with the suppressed fibroblast network without adipogenic induction (KD-FIB) and control fibroblast cells treated only with adipogenic-induction medium (NC-iADP) (adjusted *P*-value < 0.05; Figure 3A). Furthermore, several key adipocyte genes, such as PPARG, LEPR and FABP4, were significantly upregulated only in KD-iADP after 2 weeks of cultivation (Figure 3B). Gene ontology analysis further revealed that the combination of network suppression and adipogenic induction enhanced genes involved in lipid, fatty acid, coenzyme, and steroid metabolic processes, while the other conditions led to few or none of adipocyte-related genes (Figure 3C). Interestingly, KD-iADP cells also downregulated a large proportion of fibroblast genes such as TGFB2, COL5A1, FAP and MMP10 where the genes associated with developmental processes and muscle organ development were significantly suppressed (Supplementary Figure S4).

### Genome-wide expression comparison of knockdown-induced adipocytes to *bona fide* adipocytes

**Figure 4.**
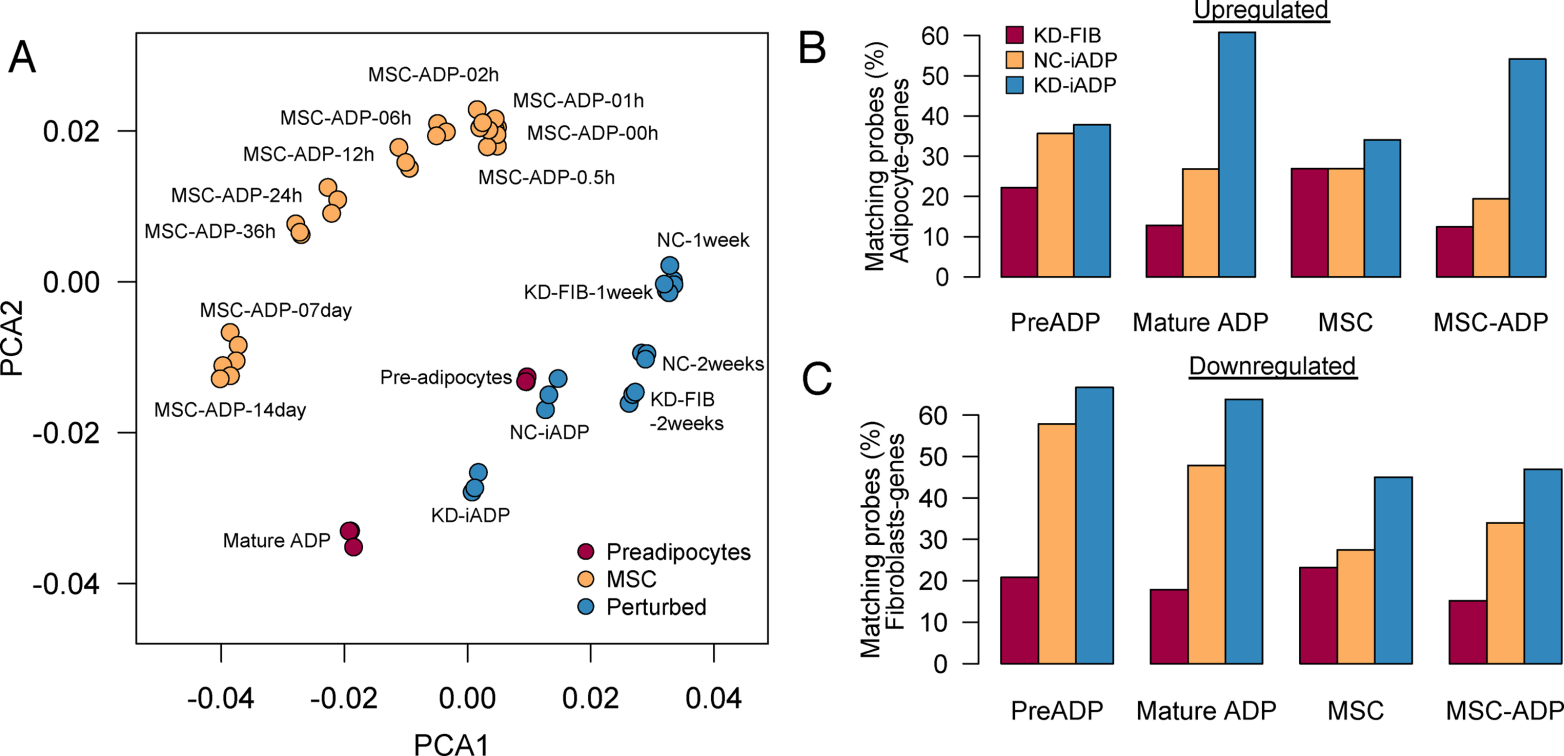
Comparison of KD-iADP cells with adipocytes. (**A**) Principal component analysis (PCA) of MSC-differentiation time course (orange), pre-adipocytes to mature adipocytes differentiation (dark red) and perturbed fibroblasts (blue) reveal that KD-iADP align with the pre-adipocyte differentiation pathway. Each circle represents a single microarray experiment and each condition is profiled in triplicates. (**B**) Quantification of upregulated genes in KD-iADP reveals significant induction of genes that are highly specific in mature adipocytes. (**C**) Conversely, large numbers of fibroblast genes were downregulated in NC-iADP and KD-iADP. The combination of knockdown and induction leads to a greater suppression of fibroblast genes.

In order to further understand the transition of KD-iADP cells relative to other sources of adipocytes, we additionally performed genome-wide transcriptional profiling including pre-adipocytes and MSCs. The principal component analysis (PCA) revealed that KD-iADP positioned in line with the pre-adipocyte differentiation pathway rather than the MSC differentiation pathway after 2 weeks (Figure 4A). These results suggest that KD-iADP may be an intermediate of pre- and mature adipocytes, indicating that a prolonged cultivation of these cells may induce a more mature form of adipocytes. In order to further assess the degree of *trans*-differentiation, we analyzed the number of differentially regulated genes for network-suppressed cells (KD-FIB), adipogenic-induced cells (NC-iADP), and both network-suppressed and adipogenic-induced (KD-iADP) cells, and compared them to genes which were highly expressed in either pre-adipocytes, mature adipocytes, or MSC and MSC-derived adipocytes when compared to fibroblasts (Figure 4B). Notably, KD-iADP cells induced more than 55% of genes expressed in mature adipocytes as well as in MSC-derived adipocytes, while a smaller portion of the induced genes overlapped with the pre-adipocytes and MSCs. It is important to note that the knockdown and the induction medium alone failed to prompt adipocyte-associated genes in fibroblasts, and that no significant MSC genes were induced, suggesting that the transition is a direct process. However, adipogenic-induction medium alone could lead to a significant downregulation of fibroblast genes when compared to pre-adipocytes, where the combined knockdown and the induction medium led to a slightly more downregulation of fibroblast genes (Figure 4C).

## DISCUSSION

In this study, we identified 18 fibroblast-enriched TFs based on the large collection of human gene expression database (FANTOM5) and inferred the TRN of human dermal fibroblasts using the Matrix-RNAi approach. We identified PPRX1, OSR1, TWIST2 and LHX9 to be highly interconnected in the fibroblastic TRN and showed that disrupting the fibroblast TRN by means of siRNA transfection allowed for the induction of adipocyte-like cells in the presence of an adipogenic medium. The KD-iADP expressed major adipocyte regulatory factors such as PPARG2 and CEBPA endogenously, while key fibroblast-specific genes were broadly downregulated after prolonged cultivation. Such a transient disruption of fibroblast TRN may be a suitable strategy for future reprogramming approaches.

Several approaches to enhance *trans*-differentiations have been published where the authors overexpressed four iPSC factors to partially reprogram fibroblasts into a ‘plastic’ state and induced *trans*-differentiation by stimulating the cells with a cocktail of small molecule compounds designed for various target cells (24). However, these models rely largely on the ectopic expression of virus-mediated transgenes, which can integrate into the genome (25–27). In contrary, we demonstrated that *trans*-differentiation could be achieved without the need of ectopic expression of transgenes. The chemical/cytokine cocktail of IBMX, indomethacin, dexamethasone and insulin was sufficient to drive adipocyte *trans*-differentiation only after the disruption of the fibroblast network.

The siRNA transfection is an ideal strategy for cell reprogramming due to their transient properties in the host cell (28,29). Interestingly, our data suggest that a prolonged cultivation of siRNA transfection (3 weeks) can maintain adipocyte-specific expression and phenotype. This suggests that the temporary suppression of the fibroblast TRN was sufficient to allow fibroblasts to overcome the cellular barrier and transit into a new state. However, because adipocytes and fibroblasts share the same mesenchymal lineage, a more stable suppression of the fibroblast TRN may promote efficient *trans*-differentiation into non-mesenchymal lineages.

Interestingly, we observed that the adipogenic-induction cocktail alone failed to induce PPARG2 expression, thus no lipid accumulation in putative fibroblast cells. PPARG2 plays a pivotal role in adipocyte differentiation and *trans*-differentiation (30). Ectopic expression of PPARG2 in conjunction with CEBPA was reported to induce a significant transformation into adipocyte-like cells in mouse embryonic fibroblasts (31–33). More recently, we observed that PPARG2 overexpression in human dermal fibroblasts induced far less adipocytes than in MSCs (11). Moreover, stable expression of fibroblastic TRN led to an inhibition of adipogenesis (Supplementary Figure S2). Collectively, this study demonstrates that the fibroblast TRN is more robust (i.e. resistant to change) than the MSC TRN, suggesting the existence of safeguard mechanisms in fully differentiated somatic cells to prevent aberrant *trans*-differentiations. Strikingly, the combination of both disruption of the fibroblast TRN and the chemical-based adipogenic induction led to endogenous activations of several key drivers of adipocyte network, namely PPARG2 and CEBPA (Figure 3B). It is possible that disrupting the fibroblastic TRN may broadly modify the epigenetic landscape of fibroblast cells, exposing the promoter regions of key adipocyte promoters, such as PPARG, for transcriptional activation (34), and only in combination with induction medium where compounds such as dexamethasone can induce wide range of epigenetic modifications and activate gene expression and differentiation (35). Thus, a synergistic role of the network disruption and the addition of chemical agents may be essential to induce transgene-free cell reprogramming.

We present the fibroblast-enriched TF set from the largest expression dataset (FANTOM5) and present the fibroblastic TRN model built by Matrix-RNAi. Moreover, we showed the first evidence that specifically targeting the TRN of fibroblasts promotes adipogenic *trans*-differentiation in the presence of a defined chemical cocktail without the need of ectopic expression. Therefore, targeting the gene regulatory network provides a novel strategy to induce or enhance cellular reprogramming pathways.

## Supplementary Data

Supplementary Data are available at NAR Online.

SUPPLEMENTARY DATA
