# Supplementary material for: A transient disruption of fibroblastic transcriptional regulatory network facilitates trans-differentiation
Source: Nucleic Acids Res. 2014 Jul 10;42(14):8905–13. doi: 10.1093/nar/gku567 (PMC4132712; doi:10.1093/nar/gku567)
Supplement: SUPPLEMENTARY DATA [file supp_42_14_8905__index.html]

A transient disruption of fibroblastic transcriptional regulatory network facilitates trans-differentiation — SUPPLEMENTARY DATA 

# A transient disruption of fibroblastic transcriptional regulatory network facilitates *trans*-differentiation

## SUPPLEMENTARY DATA

**Files in this Data Supplement:**

- Supplementary Data
