# Supplementary material for: A transient disruption of fibroblastic transcriptional regulatory network facilitates trans-differentiation
Source: Nucleic Acids Res. 2014 Jul 10;42(14):8905–13. doi: 10.1093/nar/gku567 (PMC4132712; doi:10.1093/nar/gku567)
Supplement: SUPPLEMENTARY DATA [file supp_gku567_nar-03679-v-2013-File007.zip › Supplementary_figure_2.pdf]

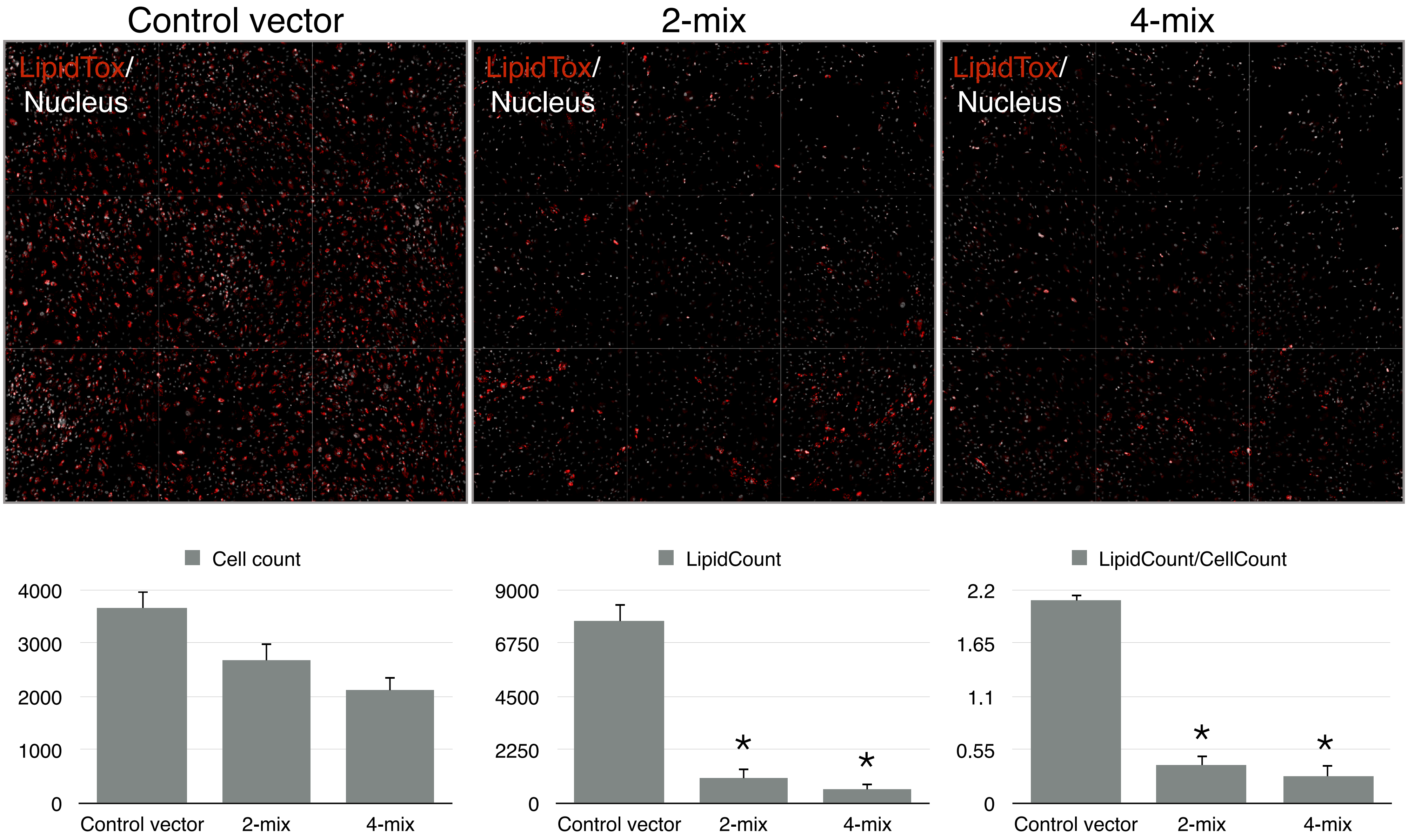

Supplementary figure 2A. Ectopic expression of fibroblastic NW inhibits adipogenesis in mesenchymal stem cells. Transcription factors PPRX1 and OSR1 (2-mix), and PPRX1, OSR1, TWIST2, LHX9 (4-mix), and empty vector control were transduced onto human mesenchymal stem cells at 5 MOI per each factor and stimulated with the adipogenic induction medium. After two weeks, cells were stained with LipidTox (red) and Hoescht (white; peudo-color) for the quantification of lipids and the total number of cells, respectively. Cells were imaged with the 5x objective lens and quantified using the Cellomics ArrayScan imager (SpotDetector protocol; ThermoScientific). The experiment was performed twice with 6 wells per each conditions. * indicates p-value less than 0.01 compared to control vector. The same experiment was imaged and quantified using the Celigo (Supplementary figure 2B).

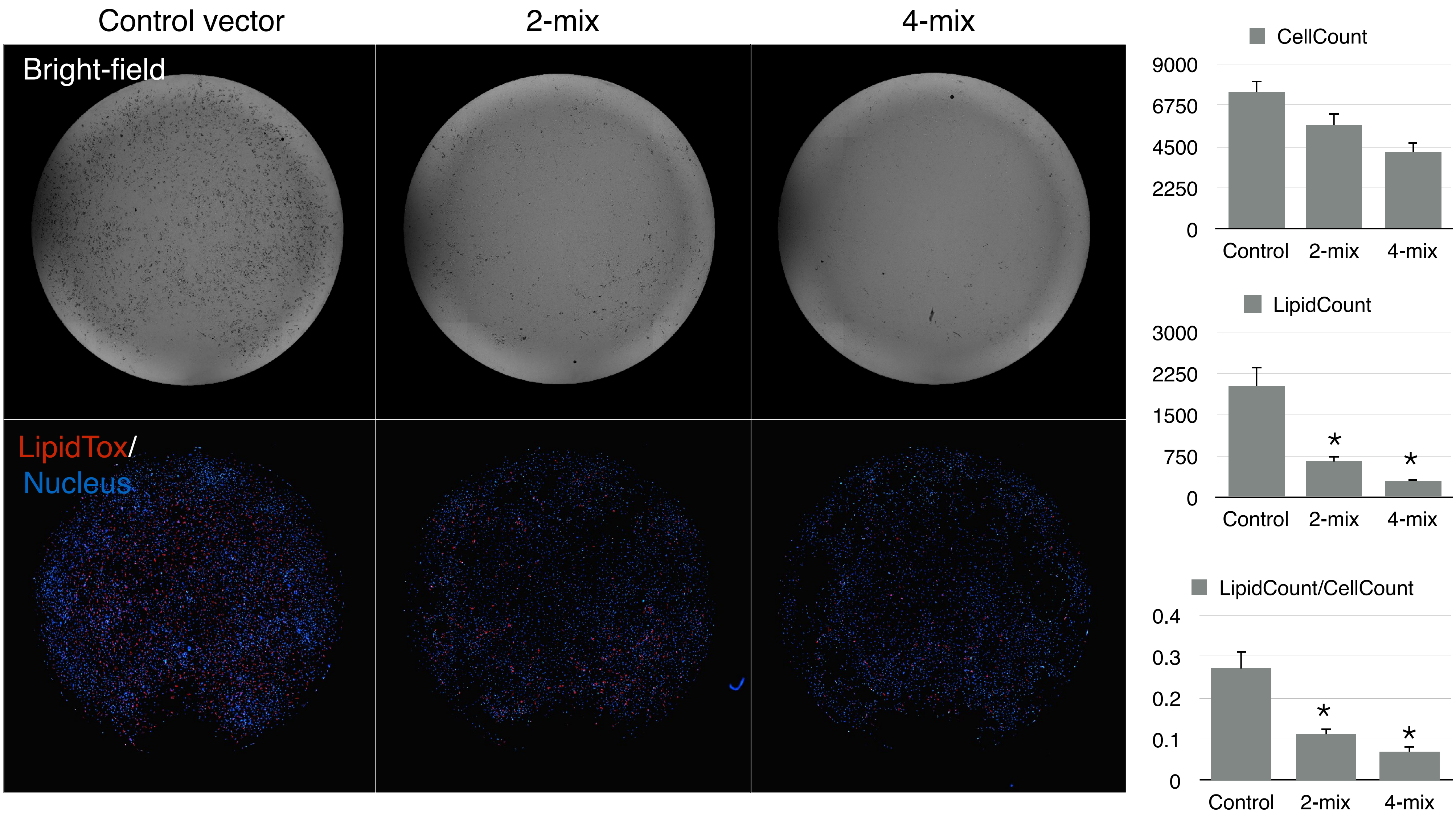

Supplementary figure 2B. Ectopic expression of fibroblastic NW inhibits adipogenesis in mesenchymal stem cells. Transcription factors PPRX1 and OSR1 (2-mix), and PPRX1, OSR1, TWIST2, LHX9 (4-mix), and empty vector control were transduced onto human mesenchymal stem cells at 5 MOI per each factor and stimulated with the adipogenic induction medium. After two weeks, cells were stained with LipidTox (red) and Hoescht (blue) for the quantification of lipids and the total number of cells, respectively. Cells were imaged using bright-field and LED based fluorescent channels (blue: ex 377/50; em 470/22, red: 531/40; em 629/53) and quantified using the Celigo S imager (Brooks). The experiment was performed twice with 6 wells per each conditions. * indicates p-value less than 0.01 compared to control vector. The same experiment was imaged and quantified using the Cellomics ArrayScan (Supplementary figure 2A).

Supplementary methods for supplementary figure 2.

Lentivirus production

Adapted from Shin JW., et al. NAR 2012 (reference no. 12). Gateway compatible human full-length cDNA entry clones derived from RIKEN BRC clone bank (http://www.brc.riken.jp/) and Invitrogen (Carlsbad, CA, USA) were recombined into 150 ng of pENTR lentivirus vector (CSII-EF-RfA-IRES2-VENUS) overnight at room temperature using Gateway LR clonaseII enzyme mix (Invitrogen). After 2 hours of Protease K treatment at 55°C, recombined plasmids were transformed into OneShot® *Stbl3* competent *E.Coli* (Invitrogen) following manufacturer's protocol. Plasmids derived from single colony were expanded and purified using PureYield Plasmid Midiprep System (Promega). For every 8.5 μg of the plasmid, 5 μg of HIV-gp and 5 μg of VSV envelop genes were co-transfected onto $4x10^6$ 293T cells (prepared the day before at 37°C, 10% CO_2) using FuGeneHD (Roche) in OPTI-MEM (WAKO) medium containing 5% FBS at 37°C 5% CO_2. Between 24h-72h of incubation, supernatant containing virus were collected and centrifuged at 19,400 RPM for 2 hours at 20°C. The pallet was then dissolved in 100 μL HBSS buffer (WAKO), followed by titer check and stored at -80°C freezer for later use.

Gene	ID	Source
OSR1	W01A002D01	RIKEN
LHX9	W02AL019B23	RIKEN
TWIST2	W01A001I24	RIKEN
PRRX1	IOH36664	Invitrogen

Virus titer check.

Adapted from Shin JW., et al. NAR 2012 (reference no. 12). One microliter of concentrated virus from the previous step was serially diluted (1:1000, 1:3000, 1:9000, 1:27000, 1:36000) in MEM-α containing 10% FBS/L-Glutamine/antibiotics (WAKO) and transduced onto 3,000 of 293T cells seeded in a black clear-bottom 96-well plate (BD) for three days. The nucleus was stained using Hoechst (Invitrogen) for 30 minutes at 37°C 5% CO_2 and the plate was subjected to Cellomics ArrayScan XTi Reader (ThermoScientific) for image analysis. FITC and Hoechst filters were used to detect Yellow Fluorescent Protein derivative (Venus), and nuclear staining, respectively, at 10x magnification, and 10 images were taken per each well. Cellomics bio-application protocol "SpotDetector" was used to segment Hoechst+ nucleus and to count Venus overlapping cells. The titer was calculated by:

Titer (c.f.u) = (dilution factor x % of Venus+ cells x initial cell number x volume per well] / volume of concentrated virus

Cell culture and virus transduction.

Mesenchymal stem cell (#PT-2501, Lonza, USA) were cultured in accordance to manufacturer's instructions. Five MOI of lentivirus was prepared for each factor and transduced with 80 mg/mL of Polybrene (Sigma) to increase transduction efficiency. Five days post virus transduction, culture medium was replaced with adipogenic induction medium for three days (IBMX, indomethacin, dexamethasone, insulin; Lonza, PT-3102B/ PT-4135) followed by maintenance medium for 4 days (insulin; Lonza, PT-3102A). The adipocyte induction regime was repeated for 1 more cycle (total two cycles).
