# Supplementary material for: A transient disruption of fibroblastic transcriptional regulatory network facilitates trans-differentiation
Source: Nucleic Acids Res. 2014 Jul 10;42(14):8905–13. doi: 10.1093/nar/gku567 (PMC4132712; doi:10.1093/nar/gku567)
Supplement: SUPPLEMENTARY DATA [file supp_gku567_nar-03679-v-2013-File007.zip › Supplementary_figure_1.pdf]

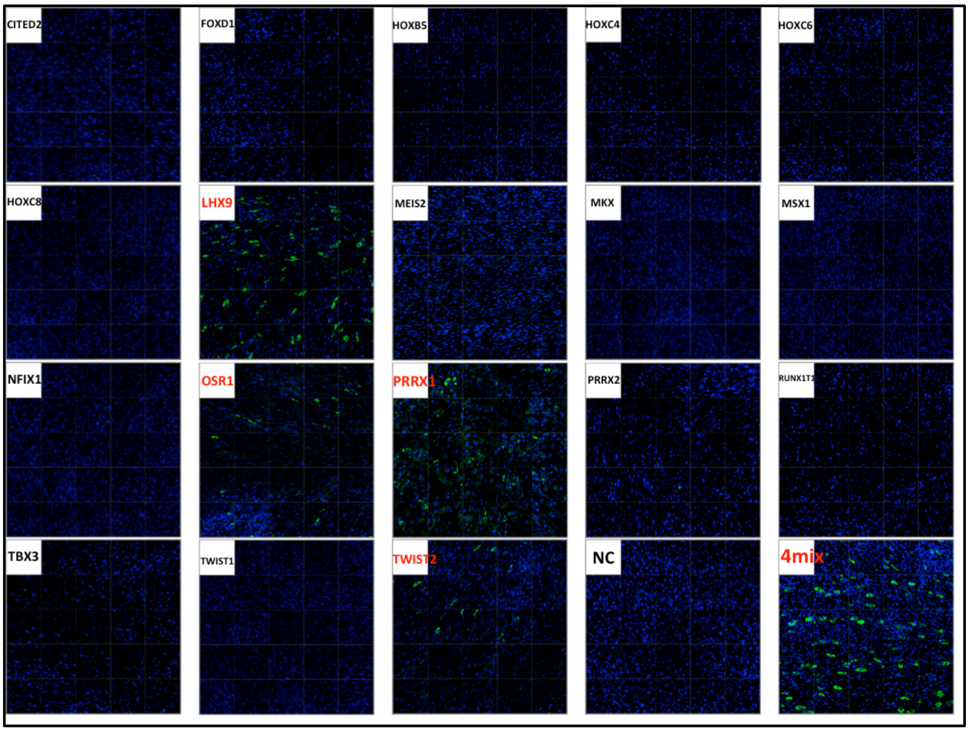

Supplementary figure1. Lipid formation in human fibroblast cells after transcription factor knockdown plus 2 weeks of adipogenesis induction regime. Nuclear and Lipid droplets were stained using Hoescht (blue) and LipidTox (Green – peudo color), respectively. 4 mix = LHX9, OSR1, PRRX1 and TWIST2 pooled siRNA. NC = scramble siRNA control. Images were taken using Cellomics ArrayScan (ThermoScientific). 25 images were taken at objective 10x magnification and merged using the Cellomics ArrayScan software.
