# Supplementary material for: A transient disruption of fibroblastic transcriptional regulatory network facilitates trans-differentiation
Source: Nucleic Acids Res. 2014 Jul 10;42(14):8905–13. doi: 10.1093/nar/gku567 (PMC4132712; doi:10.1093/nar/gku567)
Supplement: SUPPLEMENTARY DATA [file supp_gku567_nar-03679-v-2013-File007.zip › Supplementary_table_2.pdf]

Supplementary table 2. List of fibroblast-enriched transcription factors based on Illumina microarray expression profiling

	Symbol	Gene name	Gene ID	Fibroblasts-average (Log2 signal intensity)	Other cells-average (Log2 signal intensity)	Enrichment score (Microarrays)
1	HOXC8	homeobox C8	3224	11.507	7.299	4.209
2	TWIST2	twist basic helix-loop-helix transcription factor 2	13345	11.033	7.254	3.779
3	PRRX2	paired related homeobox 2	51450	11.135	7.475	3.660
4	FOXD1	forkhead box D1	297	11.964	8.340	3.624
5	HOXC6	homeobox C6	3223	12.147	8.657	3.489
6	PRRX1	paired related homeobox 1	5396	11.321	8.237	3.084
7	CITED2	Cbp/p300-interacting transactivator, with Glu/Asp-rich carboxy-terminal domain, 2	10370	12.476	9.580	2.896
8	HOXB8	homeobox B8	3218	9.012	6.190	2.822
9	MKX	mohawk homeobox	283078	9.283	6.490	2.793
10	MSX1	msh homeobox 1	4487	9.949	7.333	2.616
11	LDB2	LIM domain binding 2	9079	9.778	7.308	2.470
12	HOXB5	homeobox B5	3215	9.664	7.273	2.390
13	MEIS2	Meis homeobox 2	4212	10.197	7.940	2.257
14	HOXC4	homeobox C4	3221	9.030	6.834	2.196
15	ZNF682	zinc finger protein 682	91120	11.314	9.128	2.186
16	TCF4	transcription factor 4	6925	9.384	7.223	2.161
17	HOXC9	homeobox C9	3225	8.170	6.084	2.087
18	EMX2	empty spiracles homeobox 2	2018	8.672	6.617	2.055
19	HOXB7	homeobox B7	3217	9.185	7.189	1.996
20	RUNX1T1	runt-related transcription factor 1; translocated to, 1 (cyclin D-related)	862	8.061	6.065	1.996
21	TWIST1	twist basic helix-loop-helix transcription factor 1	7291	10.476	8.496	1.981
22	HOXB2	homeobox B2	3212	9.895	7.984	1.911
23	NFIX	nuclear factor I/X (CCAAT-binding transcription factor)	4784	11.167	9.273	1.893
24	LHX9	LIM homeobox 9	56956	7.364	5.491	1.873
25	UHRF1	ubiquitin-like with PHD and ring finger domains 1	29128	11.276	9.434	1.842
26	SBDS	Shwachman-Bodian-Diamond syndrome	51119	10.663	8.830	1.833
27	PITX2	paired-like homeodomain 2	5308	8.152	6.331	1.821
28	TCEA3	transcription elongation factor A (SII), 3	6920	9.673	7.893	1.780
29	IRX2	iroquois homeobox 2	153572	9.250	7.509	1.741
30	RUNX3	runt-related transcription factor 3	864	8.771	7.042	1.729
31	TBX1	T-box 1	6899	7.429	5.701	1.727
32	TBX3	T-box 3	6926	8.554	6.842	1.712
33	ZNF148	zinc finger protein 148	7707	10.553	8.851	1.702
34	ZNF14	zinc finger protein 14	7561	11.538	9.934	1.604
35	FOXL2	forkhead box L2	668	7.277	5.676	1.601
36	JUN	jun proto-oncogene	3725	11.569	9.976	1.593
37	LRRFIP2	leucine rich repeat (in FLII) interacting protein 2	9209	10.514	8.923	1.591
38	OSR1	odd-skipped related 1 (Drosophila)	130497	9.199	7.641	1.558
39	ZNF786	zinc finger protein 786	136051	9.659	8.108	1.551
40	PRKCDBP	protein kinase C, delta binding protein	112464	12.344	10.804	1.540
41	HOXD1	homeobox D1	3231	7.442	5.904	1.538
42	ARNT2	aryl-hydrocarbon receptor nuclear translocator 2	9915	8.162	6.630	1.532
43	TAF15	TAF15 RNA polymerase II, TATA box binding protein (TBP)-associated factor, 68kDa	8148	12.098	10.567	1.531
44	SATB2	SATB homeobox 2	23314	8.442	6.930	1.512
