# Supplementary material for: A transient disruption of fibroblastic transcriptional regulatory network facilitates trans-differentiation
Source: Nucleic Acids Res. 2014 Jul 10;42(14):8905–13. doi: 10.1093/nar/gku567 (PMC4132712; doi:10.1093/nar/gku567)
Supplement: SUPPLEMENTARY DATA [file supp_gku567_nar-03679-v-2013-File007.zip › Supplementary_figure_4.pdf]

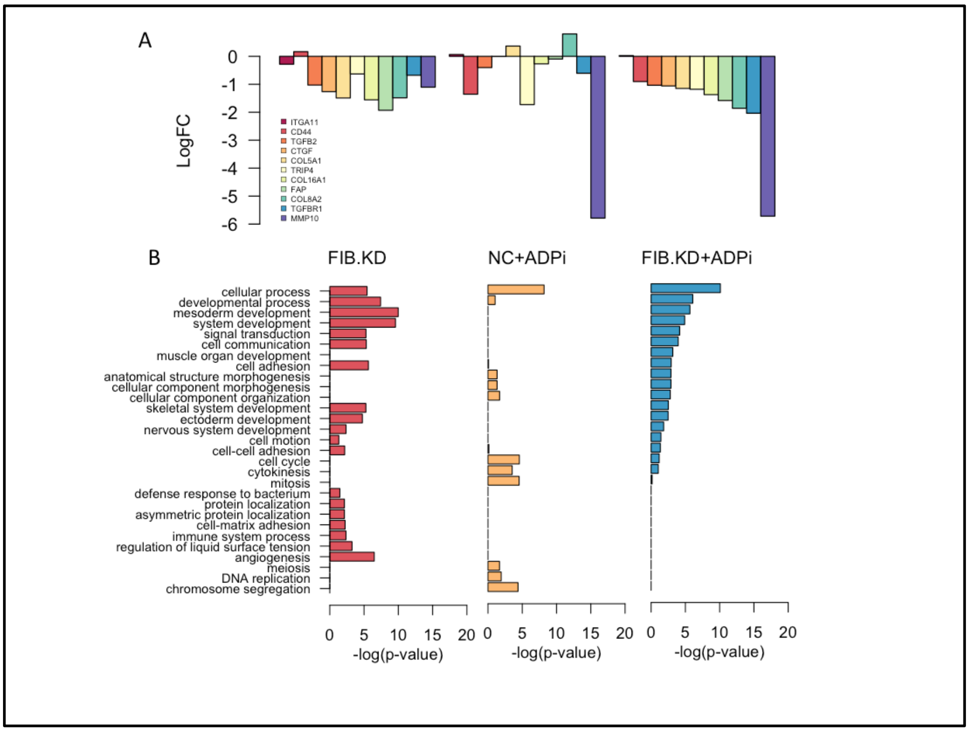

Supplementary figure 4. Expression and gene ontology analyses of downregulated genes after perturbation. (A) List of fibroblastic genes were downregulated after knockdown of fibroblastic NW ("FIB.KD"), adipogenic medium ("NC+ADPi"), and combination of both (FIB.KD+ADPi) based on qRT-PCR. Fibroblasts transfected with scramble siRNA was compared as control in all conditions. (B) Gene ontology analyses after whole transcriptome microarray profiling.
