# Supplementary material for: A transient disruption of fibroblastic transcriptional regulatory network facilitates trans-differentiation
Source: Nucleic Acids Res. 2014 Jul 10;42(14):8905–13. doi: 10.1093/nar/gku567 (PMC4132712; doi:10.1093/nar/gku567)
Supplement: SUPPLEMENTARY DATA [file supp_gku567_nar-03679-v-2013-File007.zip › Supplementary_figure_3.pdf]

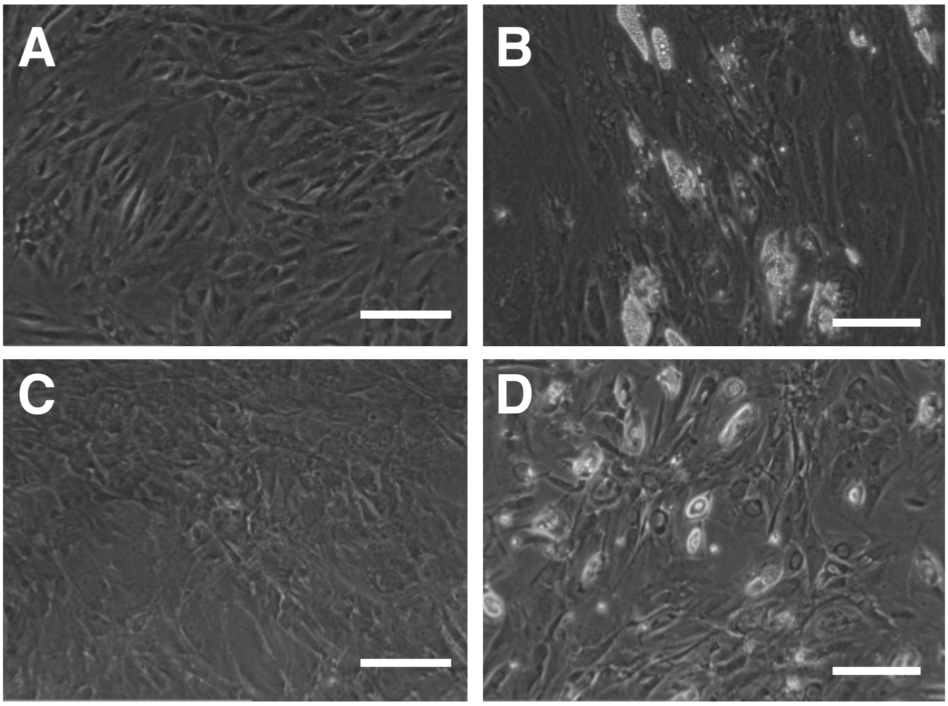

Supplementary figure 3. Human adipocytes and knockdown of FIB-network in human dermal fibroblasts. (**A**) Subcutaneous pre-adipocytes, (**B**) subcutaneous pre-adipocytes stimulated with adipogenic-induction, (**C**) siRNA negative control transfected human (adult) dermal fibroblasts stimulated with adipogenic-induction medium, (**D**) 4-TF mix knocked-down human dermal fibroblasts stimulated with adipogenic-induction medium. Scale bars = 50 μm.
